# Association of serum 25-hydroxyvitamin D levels with indicators of target organ damage in patients with diabetes: a cross-sectional study

**DOI:** 10.3389/fendo.2026.1876261

**Published:** 2026-06-29

**Authors:** Min Wang, Yanrong Huang, Yilun Gong, Lina Li, Zhenxing Zeng, Chuanfang Jiang, Guozhong Ji

**Affiliations:** 1 Department of General Practice, The Second Affiliated Hospital of Nanjing Medical University, Nanjing, Jiangsu, China; 2 Department of Endocrinology, Jen Ching Memorial Hospital, Kunshan, Jiangsu, China

**Keywords:** 25-hydroxyvitamin D, carotid plaque, diabetes, peripheral nerve damage, target organ damage

## Abstract

**Objective:**

To assess serum 25-hydroxyvitamin D [25(OH)D] in relation to indicators of target organ damage in patients with diabetes.

**Methods:**

This analysis included 372 adults with diabetes who underwent serum 25(OH)D testing in the Department of Endocrinology at Jen Ching Memorial Hospital from January 1 to December 31, 2025. Data on demographic characteristics, medical history, laboratory parameters, carotid ultrasonography, and electrophysiological examinations were collected. We performed multivariable regression models to examine the associations of serum 25(OH)D levels with peripheral nerve dysfunction, carotid plaque, urinary microalbumin (mALB), cerebral infarction, and coronary heart disease (CHD). Restricted cubic spline (RCS), threshold effect, and sensitivity analyses were further employed.

**Results:**

Serum 25(OH)D was inversely associated with peripheral nerve damage and carotid plaque. After adjustment for all prespecified covariates, the corresponding odds ratios were 0.941 (95% *CI*, 0.905–0.976; *P* = 0.001) and 0.942 (95% *CI*, 0.906–0.977; *P* = 0.002), respectively. Serum 25(OH)D levels were also inversely associated with log-transformed mALB (*β* = -0.035, 95% *CI*, -0.057 to -0.013; *P* = 0.002). In continuous-variable models, higher serum 25(OH)D was additionally associated with lower odds of cerebral infarction (*OR* = 0.896, 95% *CI*, 0.816–0.971; *P* = 0.013) and CHD (*OR* = 0.902, 95% *CI*, 0.835–0.966; *P* = 0.006) after full adjustment. RCS and exploratory threshold analyses suggested nonlinear associations of serum 25(OH)D with peripheral nerve damage and mALB. Sensitivity analyses yielded consistent results.

**Conclusions:**

Among patients with diabetes, lower serum 25(OH)D levels were associated with peripheral nerve damage, carotid plaque, and higher mALB levels. These findings support an association between serum 25(OH)D and indicators of diabetes-related target organ damage, but prospective studies are needed to clarify the temporal and clinical significance of these associations.

## 1 Introduction

Diabetes remains one of the most consequential chronic diseases worldwide, with the 11th edition of the IDF Diabetes Atlas projecting a global prevalence of 12.96% by 2050, corresponding to 853 million people (1). As the disease progresses, target organ damage involving the cardiovascular and cerebrovascular systems, the kidneys, and peripheral nerves becomes increasingly common. These complications are major contributors to disability, mortality, and healthcare burden in people with diabetes (2–4). Identifying modifiable factors associated with diabetes-related target organ damage is therefore vital for the early detection and prevention of diabetic complications.

Long viewed primarily as a regulator of mineral homeostasis and bone physiology, vitamin D is now recognized as a potential contributor to metabolic dysfunction and diabetes-related complications. Proposed mechanisms include effects on pancreatic β-cell function, insulin sensitivity, and immune regulation (5–7). Circulating 25-hydroxyvitamin D [25(OH)D] is generally regarded as the standard indicator for evaluating vitamin D status. Interest in the association between serum 25(OH)D levels and diabetes-related target organ damage has gained increasing attention.

Previous work has generally reported an inverse relationship between serum 25(OH)D concentrations and diabetic neuropathy or vascular complications. Observational data further suggest that vitamin D deficiency is associated with a greater likelihood of diabetic peripheral neuropathy (8, 9). In hospitalized patients with type 2 diabetes, vitamin D deficiency has also been independently associated with subclinical distal symmetric polyneuropathy (10). A systematic review and meta-analysis further found an association between lower vitamin D levels and greater cardiovascular disease risk in patients with diabetes (11). However, the available evidence remains inconsistent. More recent Mendelian randomization analyses did not support a significant causal relationship between vitamin D levels and diabetic neuropathy, diabetic kidney disease, or diabetic retinopathy (12–14).

Overall, the relationship of serum 25(OH)D in diabetes-related target organ damage, particularly neural and vascular injury, remains insufficiently characterized. Given the growing clinical importance of early identification of diabetic complications and risk stratification in chronic disease management, clarifying this association may have clinical relevance.

Accordingly, we evaluated serum 25(OH)D levels in relation to indicators of diabetes-related target organ damage, seeking to inform risk stratification and clinical decision-making in diabetes care.

## 2 Methods

### 2.1 Study population

We analyzed 372 inpatients with type 1 or type 2 diabetes mellitus who were treated at the Department of Endocrinology, Jen Ching Memorial Hospital, from January 1 to December 31, 2025, in a hospital-based cross-sectional study. Eligibility required measurement of serum 25(OH)D during hospitalization. The analysis was restricted to hospitalized patients because these patients routinely underwent serum 25(OH)D testing as well as comprehensive clinical, laboratory, and diabetes-related complication assessments during hospitalization, allowing a more standardized evaluation of diabetes-related target organ damage and reducing missing key data. Our protocol was reviewed and approved by the institutional ethics committee of Jen Ching Memorial Hospital (approval No. 202501), and all participants provided written informed consent.

This study was reported in accordance with the Strengthening the Reporting of Observational Studies in Epidemiology (STROBE) statement for cross-sectional studies. The completed STROBE checklist is provided as **Supplementary File 1**.

### 2.2 Patient eligibility

#### 2.2.1 Eligibility requirements

The analysis included only patients who fulfilled all prespecified requirements: (1) adults aged 18 years or above; (2) established diagnosis of type 1 or type 2 diabetes mellitus; (3) serum 25(OH)D measurement during hospitalization; and (4) availability of complete major clinical and laboratory data.

#### 2.2.2 Reasons for exclusion

Patients were not considered for analysis if any of the following applied: (1) use of renin–angiotensin–aldosterone system inhibitors, including angiotensin-converting enzyme inhibitors or angiotensin receptor blockers; (2) gestational diabetes or another specific form of diabetes; (3) severe hepatic or renal dysfunction; (3) active tuberculosis, malignant tumors, autoimmune diseases, or severe psychiatric disorders; (5) acute diabetic complications within the previous month; (6) acute infection, acute stress, or other acute conditions that could markedly affect vitamin D levels or metabolic status; or (7) missing key clinical data or primary outcome data.

### 2.3 Data collection

Demographic information, clinical characteristics, medical history, and laboratory results were extracted by reviewing electronic medical records. Demographic and clinical characteristics included age, gender, body mass index (BMI), smoking history, alcohol consumption, diabetes type, diabetes duration, type of vitamin D supplementation, and glucose-lowering treatment during hospitalization.

Medical history included hypertension, non-alcoholic fatty liver disease (NAFLD), coronary heart disease (CHD), and cerebral infarction. Fasting blood and urine specimens were obtained on the morning of the second hospital day. The following variables were measured: serum 25(OH)D, glycated hemoglobin A1c (HbA1c), fasting C-peptide, uric acid (UA), creatinine (Cr), serum calcium, triglycerides (TG), total cholesterol (TC), low-density lipoprotein cholesterol (LDL-C), urinary microalbumin (mALB), and seven thyroid function parameters.

Carotid ultrasonography and electrophysiological examinations were performed during hospitalization, and the results were determined from the formal reports. Two trained investigators independently extracted the data and verified the entries through cross-checking. Vitamin D deficiency, insufficiency, and sufficiency were defined as serum 25(OH)D concentrations of < 20 ng/mL, 20 to < 30 ng/mL, and ≥ 30 ng/mL, respectively (15).

Serum 25(OH)D was measured using a chemiluminescence immunoassay on a Zhengzhou Autobio A6200 chemiluminescence analyzer. Peripheral neuropathy and carotid plaque were determined according to the formal electrophysiological examination and carotid ultrasound reports, respectively. **Supplementary Methods S1** provides detailed diagnostic criteria, outcome definitions, assay standardization procedures, laboratory measurement methods, missing data handling procedures, multicollinearity diagnostics, and model formulas with variable coding and transformations.

### 2.4 Statistical methods

Analyses were run in R v4.5.1. Continuous data were reported as mean ± standard deviation or median, according to distribution, and compared across groups with one-way analysis of variance or the Kruskal–Wallis rank-sum test. Categorical data were reported as n (%) and analyzed with the chi-square test or Fisher’s exact test when appropriate. Serum 25(OH)D level was treated as the primary study variable. Logistic regression models were fitted for peripheral nerve damage, carotid plaque, CHD, and cerebral infarction. A linear regression model was fitted for log-transformed mALB. Serum 25(OH)D was analyzed both continuously and categorically.

Four models were constructed in the multivariable regression analyses. The crude model was unadjusted. Model 1 was adjusted for age, gender, and BMI. Model 2 was further adjusted for smoking history, alcohol consumption, diabetes type, and diabetes duration. Model 3 was further adjusted for hypertension and NAFLD. Logistic regression results were expressed as odds ratios (*OR*s), and linear regression results as regression coefficients (*β*), both with 95% confidence intervals (95% *CI*s).

Restricted cubic splines (RCS) were used to examine dose–response patterns between serum 25(OH)D concentrations and each outcome. Four knots were specified, and tests for nonlinearity were used. In sensitivity analyses, the main models were further refitted after additional adjustment for UA, Cr, HbA1c, fasting C-peptide, glucose-lowering treatment, the seven-item thyroid function panel, type of vitamin D supplementation, TC, TG, and LDL-C. A two-sided *P* value < 0.05 was considered statistically significant.

To assess the potential impact of selection related to angiotensin-converting enzyme inhibitors (ACEIs) or angiotensin receptor blockers (ARBs) on the mALB findings, an additional sensitivity analysis was performed by re-including the 40 patients who had previously been excluded because of ACEI or ARB use. In this expanded analysis set, ACEI/ARB use was additionally adjusted for as a covariate based on Model 3.

No adjustment for multiple comparisons was applied to the primary exploratory analyses; a Bonferroni correction for the five primary outcomes was performed as a sensitivity analysis (**Supplementary Table 1**). Threshold effect analyses were regarded as *post hoc* exploratory and hypothesis-generating.

## 3 Results

### 3.1 Baseline characteristics of the study population

A total of 372 patients with diabetes were included in this study. The median serum 25(OH)D concentration was 18.69 (15.80, 22.42) ng/mL. Based on serum 25(OH)D levels, 25 participants were categorized as sufficient, 133 as insufficient, and 214 as deficient.

Comparisons among the three 25(OH)D groups showed significant differences in age, gender, cerebral infarction, carotid plaque, peripheral nerve damage, and type of vitamin D supplementation (all *P* < 0.05). The deficiency group was younger than the sufficiency and insufficiency groups, included more women, and had higher prevalences of carotid plaque, peripheral nerve damage, and cerebral infarction. No significant differences were observed in other demographic characteristics, metabolic indicators, complication history, or treatment-related variables (all *P* > 0.05; **Table 1**; **Supplementary Table 2**).

**Table 1:** Baseline characteristics included in multivariable adjustment models according to serum 25(OH)D status.

Characteristics	25(OH)D levels				
	All (N = 372)	Sufficiency (N = 25)	Insufficiency (N = 133)	Deficiency (N = 214)	*P* value
Age, (years)	50.23 ± 13.48	55.12 ± 11.43	51.42 ± 11.62	48.93 ± 14.58	0.031
Sex, n (%)					< 0.001
female	92 (24.73%)	1 (4.00%)	23 (17.29%)	68 (31.78%)	
male	280 (75.27%)	24 (96.00%)	110 (82.71%)	146 (68.22%)	
BMI, n (%)					0.323
<24	134 (36.02%)	11 (44.00%)	55 (41. 35%)	68 (31.78%)	
24-27.9	147 (39.52%)	10 (40.00%)	49 (36.84%)	88 (41.12%)	
>=28	91 (24.46%)	4 (16.00%)	29 (21.80%)	58 (27.10%)	
Smoking, n (%)					0.390
No	226 (60.75%)	12 (48.00%)	81 (60.90%)	133 (62.15%)	
Yes	146 (39.25%)	13 (52.00%)	52 (39.10%)	81 (37.85%)	
Alcohol use, n (%)					0.536
No	270 (72.58%)	17 (68.00%)	93 (69.92%)	160 (74.77%)	
Yes	102 (27.42%)	8 (32.00%)	40 (30.08%)	54 (25.23%)	
Diabetes type, n (%)					0.118
T1DM	26 (6.99%)	1 (4.00%)	5 (3.76%)	20 (9.35%)	
T2DM	346 (93.01%)	24 (96.00%)	128 (96.24%)	194 (90.65%)	
Diabetes duration, (years)	5 (1.00, 10.00)	6 (1.00, 10.00)	5 (1.00, 10.00)	4 (1.00, 9.00)	0.523
Hypertension, n (%)					0.644
No	241 (64.78%)	15 (60.00%)	90 (67.67%)	136 (63.55%)	
Yes	131 (35.22%)	10 (40.00%)	43 (32.33%)	78 (36.45%)	
NAFLD, n (%)					0.195
No	118 (31.72%)	11 (44.00%)	46 (34.59%)	61 (28.50%)	
Yes	254 (68.28%)	14 (56.00%)	87 (65.41%)	153 (71.50%)	

Values are presented as mean ± SD, median (Q1, Q3), or n (%), as appropriate. Age was normally distributed and compared using one-way ANOVA, whereas diabetes duration was non-normally distributed and compared using the Kruskal–Wallis test. Categorical variables were compared using the chi-square test or Fisher’s exact test, as appropriate. Normality was assessed using the Shapiro–Wilk test; NAFLD, non-alcoholic fatty liver disease.

### 3.2 Associations between serum 25(OH)D levels and major target organ outcomes

#### 3.2.1 Peripheral nerve damage

Logistic regression analyses indicated an independent inverse relationship between serum 25(OH)D and peripheral nerve damage. When 25(OH)D was entered into the model as a continuous variable, this association was observed in both the crude and multivariable-adjusted models. In Model 3, each 1 ng/mL increase in 25(OH)D corresponded to approximately 5.90% lower odds of peripheral nerve damage (*OR* = 0.941, 95% *CI*, 0.905–0.976; *P* = 0.001).

**Figure 1:**
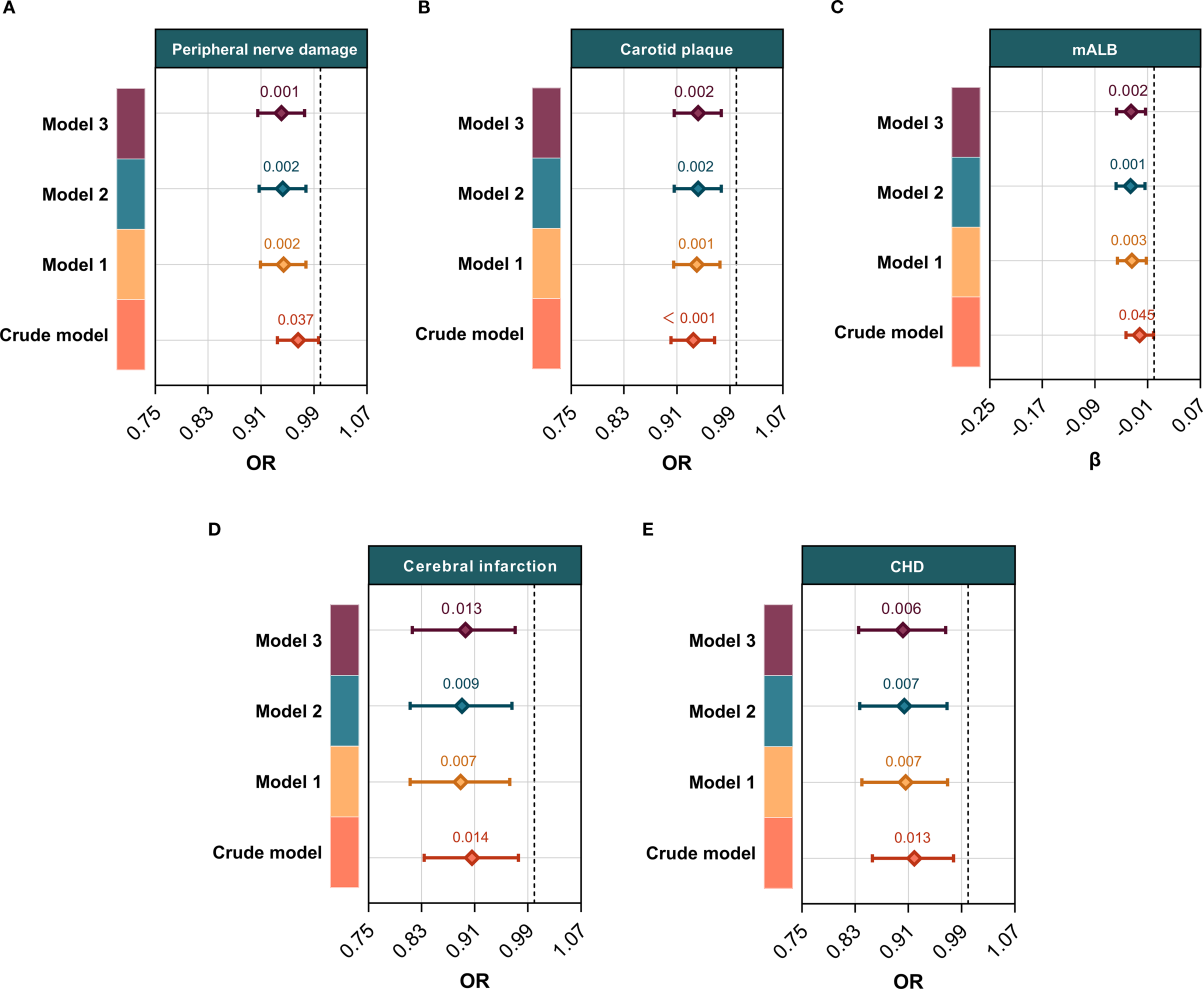
Forest plots of the associations between serum 25(OH)D levels and five target organ damage indicators across different regression models. **(A)** Peripheral nerve damage **(B)** Carotid plaque **(C)** urinary microalbumin (mALB) **(D)** Cerebral infarction **(E)** Coronary heart disease (CHD). For each outcome, results from the crude model, Model 1, Model 2, and Model 3 are presented. Peripheral nerve damage, carotid plaque, cerebral infarction, and CHD are presented as *ORs* (95% CIs), with the reference line at *OR* = 1; ln(mALB) is presented as β coefficients (95% CIs). The crude model was unadjusted. Model 1 adjusted for age, sex, and BMI. Model 2 further adjusted for smoking, alcohol use, diabetes type, and diabetes duration. Model 3 further adjusted for hypertension and NAFLD.

In the categorical analysis, the sufficiency group was used as the reference. The insufficiency group did not differ significantly from the reference group, whereas the deficiency group tended to have higher odds of peripheral nerve damage, although the between-group difference was not statistically significant. However, trend tests were significant in all models, indicating an overall increase in the risk of peripheral nerve damage with lower 25(OH)D levels (**Table 2**; **Figure 1A**).

**Table 2:** Associations between serum 25(OH)D levels and peripheral nerve damage, carotid plaque, and mALB.

Outcome	Crude model estimate (95% *CI*)	*P* value	Model 1 estimate (95% *CI*)	*P* value	Model 2 estimate (95% *CI*)	*P* value	Model 3 estimate (95% *CI*)	*P* value
Peripheral nerve damage								
25(OH)D (continuous)	0.966 (0.935, 0.997)	0.037	0.944 (0.909, 0.978)	0.002	0.943 (0.907, 0.978)	0.002	0.941 (0.905, 0.976)	0.001
Sufficiency	Ref		Ref		Ref		Ref	
Insufficiency	0.548 (0.223, 1.295)	0.175	0.665 (0.260, 1.644)	0.380	0.653 (0.248, 1.669)	0.377	0.636 (0.240, 1.639)	0.352
Deficiency	1.431 (0.595, 3.318)	0.408	2.398 (0.934, 6.049)	0.064	2.365 (0.893,6.161)	0.078	2.554 (0.955, 6.728)	0.058
*P* for trend	1.719 (1.230, 2.419)	0.002	2.284 (1.569, 3.371)	< 0.001	2.273 (1.541, 3.402)	< 0.001	2.411 (1.623, 3.640)	< 0.001
Carotid plaque								
25(OH)D (continuous)	0.935(0.901, 0.967)	< 0.001	0.940 (0.905, 0.975)	0.001	0.942 (0.906, 0.977)	0.002	0.942 (0.906, 0.977)	0.002
Sufficiency	Ref		Ref		Ref		Ref	
Insufficiency	1.143 (0.464, 2.716)	0.765	1.024 (0.411, 2.464)	0.958	1.022 (0.407, 2.476)	0.961	1.033 (0.411, 2.510)	0.943
Deficiency	2.435 (0.999, 5.729)	0.043	2.059 (0.820, 5.005)	0.114	1.995 (0.789, 4.888)	0.134	1.991 (0.785, 4.900)	0.137
*P* for trend	1.779 (1.247, 2.546)	0.002	1.653 (1.138, 2.410)	0.008	1.615 (1.106, 2.366)	0.013	1.605 (1.096, 2.358)	0.015
mALB								
25(OH)D (continuous)	-0.022 (-0.043, -0.001)	0.045	-0.034 (-0.056, -0.012)	0.003	-0.036 (-0.058, -0.014)	0.001	-0.035 (-0.057, -0.013)	0.002
Sufficiency	Ref		Ref		Ref		Ref	
Insufficiency	-0.267 (-0.854, 0.320)	0.371	-0.168 (-0.751, 0.416)	0.573	-0.120 (-0.773, 0.374)	0.494	-0.191 (-0.763, 0.381)	0.511
Deficiency	0.017 (-0.552, 0.586)	0.954	0.232 (-0.347, 0.810)	0.432	0.243 (-0.326, 0.812)	0.402	0.220 (-0.348, 0.789)	0.446
*P* for trend	0.139 (-0.087, 0.364)	0.227	0.250 (0.017, 0.483)	0.036	0.272 (0.041, 0.502)	0.021	0.250 (0.020, 0.481)	0.034

Crude model, unadjusted. Model 1 adjusted for age, sex, and BMI. Model 2 further adjusted for smoking, alcohol use, diabetes type, and diabetes duration. Model 3 further adjusted for hypertension and NAFLD. *OR*, odds ratio; *CI*, confidence interval; Odds ratios (*OR*s) were estimated for peripheral nerve damage and carotid plaque, while *β* coefficients were estimated for ln(mALB).

#### 3.2.2 Carotid plaque

Serum 25(OH)D was also negatively related to carotid plaque. When 25(OH)D was treated as a continuous measure, the crude model yielded an *OR* of 0.935 (95% *CI*, 0.901–0.967; *P* < 0.001). The estimate changed little after adjustment for potential covariates, with an *OR* of 0.942 (95% *CI*, 0.906–0.977; *P* = 0.002) in Model 3.

In the categorical analysis, with the sufficiency group as the reference, the *OR*s for the deficiency group were greater than 1 across all models, although statistical significance was reached only in the crude model. The associations were no longer statistically significant after multivariable adjustment. Trend tests showed that the likelihood of carotid plaque increased as 25(OH)D levels decreased (all *P* < 0.05) (**Table 2**; **Figure 1B**).

#### 3.2.3 Urinary microalbumin

Linear regression models were fitted using log-transformed mALB as the dependent variable. When 25(OH)D was modeled continuously, higher serum 25(OH)D was associated with lower mALB in the crude model (*β* = -0.022, 95% *CI*, -0.043 to -0.001; *P* = 0.045). This inverse relationship persisted after stepwise adjustment for covariates. In Model 3, each 1 ng/mL increase in 25(OH)D corresponded to a mean decrease of 0.035 in ln(mALB) (*β* = -0.035, 95% *CI*, -0.057 to -0.013; *P* = 0.002).

When 25(OH)D was analyzed as a categorical variable, neither the insufficiency group nor the deficiency group differed significantly from the sufficiency group. However, trend tests were statistically significant in Models 1, 2, and 3, but not in the crude model, suggesting an overall inverse association between 25(OH)D categories and ln(mALB) (**Table 2**; **Figure 1C**).

#### 3.2.4 Cerebral infarction and coronary heart disease

For cerebral infarction and CHD, logistic regression models with 25(OH)D entered as a continuous variable showed inverse associations with both outcomes. After full adjustment, each 1 ng/mL increase in serum 25(OH)D was associated with lower odds of cerebral infarction (*OR* = 0.896, 95% *CI*, 0.816–0.971; *P* = 0.013) and CHD (*OR* = 0.902, 95% *CI*, 0.835–0.966; *P* = 0.006).

Given the limited sample size of the 25(OH)D sufficiency group and the absence of cerebral infarction and CHD events in this group, categorical analyses for these outcomes were unstable and were therefore not presented. Accordingly, only continuous 25(OH)D analyses are reported for cerebral infarction and CHD. The corresponding categorical *OR*s and *P* for trend values were not reported to avoid potentially misleading interpretation. (both *P* < 0.05, **Table 3**; **Figures 1D, E**).

**Table 3:** Associations between serum 25(OH)D levels and cerebral infarction and CHD.

Outcome	Crude model *OR* (95% *CI*)	*P* value	Model 1 *OR* (95% *CI*)	*P* value	Model 2 *OR* (95% *CI*)	*P* value	Model 3 *OR* (95% *CI*)	*P* value
Cerebral infarction								
25(OH)D (continuous)	0.906 (0.834, 0.976)	0.014	0.889 (0.813, 0.963)	0.007	0.891 (0.813, 0.966)	0.009	0.896 (0.816, 0.971)	0.013
CHD								
25(OH)D (continuous)	0.919 (0.856, 0.978)	0.013	0.906 (0.840, 0.969)	0.007	0.904 (0.837, 0.968)	0.007	0.902 (0.835, 0.966)	0.006

Crude model, unadjusted. Model 1 adjusted for age, sex, and BMI. Model 2 further adjusted for smoking, alcohol use, diabetes type, and diabetes duration. Model 3 further adjusted for hypertension and NAFLD. CHD, coronary heart disease; *OR*, odds ratio; *CI*, confidence interval.

As an exploratory sensitivity analysis, the sufficiency and insufficiency groups were combined into a non-deficient group (25(OH)D ≥ 20 ng/mL) and compared with the deficiency group (25(OH)D < 20 ng/mL) using Fisher’s exact test. The results are provided in **Supplementary Table 3** and should be interpreted with caution because of the limited number of events and low statistical power.

### 3.3 Dose-response relationship analysis

**Figure 2:**
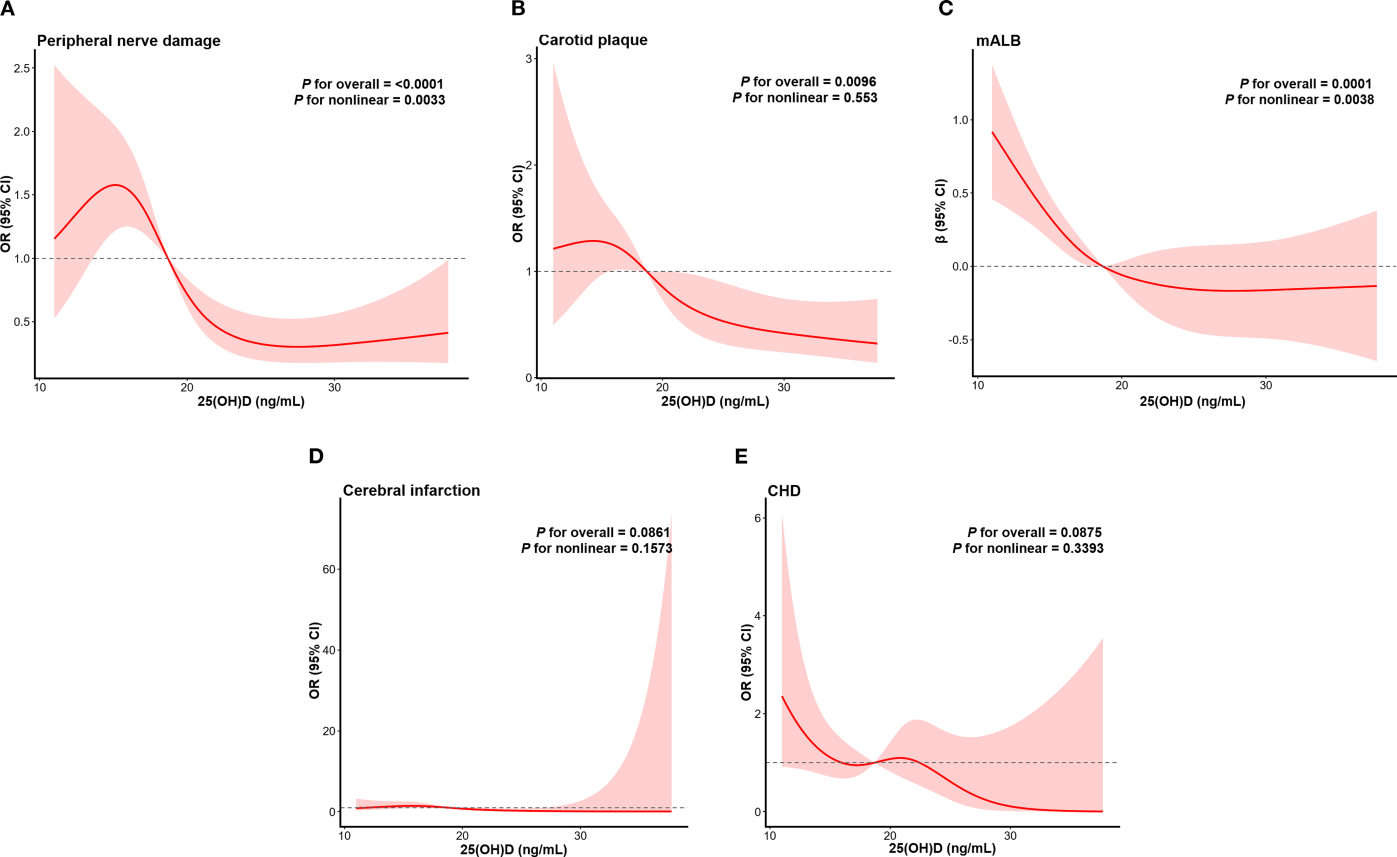
Restricted cubic spline analyses of the associations between serum 25(OH)D levels and target organ damage indicators. **(A)** Peripheral nerve damage **(B)** Carotid plaque **(C)** urinary microalbumin (mALB) **(D)** Cerebral infarction **(E)** Coronary heart disease (CHD). Restricted cubic spline analyses were performed using four knots placed at the 5th, 35th, 65th, and 95th percentiles of serum 25(OH)D. The red line represents the adjusted effect estimate, and the shaded area represents the 95% confidence interval. Peripheral nerve damage, carotid plaque, cerebral infarction, and CHD are presented as ORs (95% *CI*s), with the dashed line indicating the reference line at *OR* = 1; mALB is presented as *β* coefficients (95% *CI*s), with the dashed line indicating the reference line at *β* = 0. The restricted cubic spline models were adjusted for the same covariates as Model 3: age, sex, BMI, smoking, alcohol use, diabetes type, diabetes duration, hypertension, and NAFLD.

RCS modeling suggested nonlinear associations of serum 25(OH)D with mALB (*P* for nonlinear = 0.0038) and peripheral nerve damage (*P* for nonlinear = 0.0033) (**Figure 2**). In contrast, the association with carotid plaque was significant overall (*P* for overall = 0.0096), but showed no evidence of nonlinearity (*P* for nonlinear = 0.553).

Threshold effect analyses were then performed. As shown in **Table 4**, when serum 25(OH)D was < 22.94 ng/mL, each 1 ng/mL increase in 25(OH)D was associated with a 12.50% lower odds of peripheral nerve damage (*OR* = 0.875, 95% *CI*, 0.817–0.938; *P* < 0.001). No significant association was found at concentrations of 22.94 ng/mL or higher (*P* = 0.971), indicating that the association was largely limited to the lower range of 25(OH)D.

**Table 4:** Threshold effect analyses of serum 25(OH)D for peripheral nerve damage and mALB.

Outcome	Threshold (ng/mL)	Segment of 25(OH)D	Estimate (95% *CI*)	*P* value
Peripheral nerve damage	22.94	< 22.94	*OR* = 0.875 (0.817, 0.938)	*P* < 0.001
		≥ 22.94	*OR* = 0.999 (0.941, 1.060)	*P* = 0.971
mALB	11.48	< 11.48	*β* = -0.610 (-0.925, -0.296)	*P* < 0.001
		≥11.48	*β* = -0.025 (-0.047, -0.003)	*P* = 0.028

*OR*s were estimated for peripheral nerve damage, and *β* coefficients were estimated for ln(mALB). Threshold effect analyses were adjusted for the same covariates as Model 3. *β* coefficients in the threshold-effect analysis represent segment-specific changes in ln(mALB) per 1 ng/mL increase in serum 25(OH)D within each segment. The threshold values were data-driven and derived from the same dataset without internal validation, such as bootstrapping; therefore, they should be interpreted as exploratory and hypothesis-generating only and should not be considered fixed cutoffs.

For mALB, each 1 ng/mL increase in 25(OH)D was associated with a mean decrease of 0.610 in ln(mALB) below 11.48 ng/mL (*β* = -0.610, 95% *CI*, -0.925 to -0.296; *P* < 0.001). Above this threshold, the inverse association remained significant but was much weaker (*β* = -0.0248, 95% *CI*, -0.0468 to -0.0027; *P* = 0.028), suggesting a stronger effect at lower 25(OH)D concentrations.

These thresholds were derived from the same dataset without internal validation, such as bootstrapping, and therefore should not be interpreted as fixed cutoffs or definitive thresholds.

### 3.4 Sensitivity analyses and model diagnostics

On the basis of the fully adjusted models, further adjustment for UA, HbA1c, fasting C-peptide, Cr, glucose-lowering treatment, thyroid function parameters, type of vitamin D supplementation, TG, TC, and LDL-C did not materially alter the direction of the associations between serum 25(OH)D and peripheral nerve damage, carotid plaque, mALB, cerebral infarction, or CHD. All associations remained statistically significant (all *P* < 0.05), supporting the robustness of the main findings (**Table 5**).

**Table 5:** Sensitivity analyses for the associations between serum 25(OH)D and target organ damage indicators.

Outcome	Estimate (95% *CI*)	*P* value
Peripheral nerve damage	*OR* = 0.915 (0.865, 0.964)	0.001
Carotid plaque	*OR* = 0.928 (0.876, 0.979)	0.009
mALB	*β* = -0.040 (-0.067, -0.012)	0.004
Cerebral infarction	*OR* = 0.883 (0.784, 0.988)	0.036
CHD	*OR* = 0.903 (0.819, 0.988)	0.036

Based on Model 3, the sensitivity analysis further adjusted for uric acid, HbA1c, fasting C-peptide, creatinine, treatment regimen, thyroid function, vitamin D supplementation type, triglycerides, total cholesterol, and LDL-C; *OR*, odds ratio; *CI*, confidence interval.

In an additional sensitivity analysis, the 40 patients previously excluded because of ACEI or ARB use were re-included, resulting in an expanded analysis set of 412 patients. The proportions of ACEI/ARB use were 10.71%, 10.14%, and 9.32% in the 25(OH)D sufficiency, insufficiency, and deficiency groups, respectively, with no statistically significant between-group difference (*P* = 0.895; **Supplementary Table 4**). After additional adjustment for ACEI/ARB use based on Model 3, the inverse association between serum 25(OH)D and ln(mALB) remained directionally consistent and statistically significant, although the effect estimate was slightly attenuated (*β* = -0.030, 95% *CI*, -0.053 to -0.007; *P* = 0.010; **Supplementary Table 5**).

Because this was a retrospective cross-sectional study, no pre-specified sample size calculation was performed. *Post hoc* power estimates for the primary continuous 25(OH)D analyses of peripheral nerve damage, carotid plaque, and ln(mALB) are provided for descriptive purposes only in **Supplementary Table 6** and should not be interpreted as evidence of adequate study design or that the study was adequately powered.

Model diagnostic analyses are provided in the **Supplementary Methods S1**. No substantial multicollinearity was observed in the fully adjusted models, with all adjusted VIF values below 5. Residual diagnostic plots for the ln(mALB) model showed no obvious nonlinear pattern, although mild heteroscedasticity and tail departures from normality were observed. Apparent calibration plots for the logistic regression models showed generally acceptable agreement between predicted probabilities and observed event rates. In addition, Firth penalized logistic regression for cerebral infarction and CHD yielded results consistent with the ordinary logistic regression models.

## 4 Discussion

This cross-sectional analysis simultaneously assessed the relationships between serum 25(OH)D concentrations and peripheral nerve damage, carotid plaque, mALB, cerebral infarction, and CHD in individuals with diabetes. The main findings were further examined using restricted cubic spline analyses, threshold effect analyses, and sensitivity analyses.

It is worth noting that HbA1c did not differ significantly across the 25(OH)D sufficiency, insufficiency, and deficiency groups. This observation suggests that the associations between serum 25(OH)D and target organ damage indicators may not be explained solely by differences in chronic glycemic control. In addition, further adjustment for HbA1c in the sensitivity analyses did not materially change the direction or statistical significance of the associations. These findings support the biological plausibility that the associations of 25(OH)D with target organ damage indicators may involve neurovascular and microvascular pathways not fully captured by HbA1c. Nevertheless, causality cannot be inferred from this cross-sectional study.

Considering the involvement of vitamin D in glycemic regulation, inflammatory pathways, and neurovascular injury, its relationship with diabetes-related target organ damage is likely to be complex. Lower serum 25(OH)D concentrations were associated with higher odds of peripheral nerve damage, broadly consistent with previous reports. Sun et al. (10) documented an independent relationship between vitamin D deficiency and subclinical distal symmetric polyneuropathy among inpatients with type 2 diabetes. Consistently, a meta-analysis by Yammine et al. (16) showed a higher prevalence of vitamin D deficiency in diabetic individuals with neuropathy. The 2025 American Diabetes Association Standards of Care in Diabetes (17) identify diabetic neuropathy as an important chronic complication that impairs functional status and quality of life.

Several mechanisms may underlie the association between 25(OH)D and peripheral nerve damage. Vitamin D may mitigate upstream processes involved in nerve injury, including glucotoxicity, inflammation, and oxidative stress, by improving pancreatic β-cell function and insulin responsiveness (18, 19). In parallel, vitamin D may contribute to the maintenance and repair of peripheral nerves through vitamin D receptor (VDR)-related signaling, promote neurotrophic factor expression (20, 21), improve nerve fiber function, and attenuate inflammatory and oxidative injury to neural tissue and Schwann cells under hyperglycemic conditions (22–24). In addition, diabetic peripheral neuropathy is, at least in part, a neural microvascular disorder, and vitamin D may also be involved through its role in endothelial function and microcirculation (25). Together, these pathways offer a mechanistic basis for the relationship identified in our analysis.

Notably, beyond these metabolic, inflammatory, and neurotrophic pathways, our data suggested that the association between 25(OH)D and peripheral nerve damage may not be simply linear. The exploratory threshold analysis identified an inflection point at approximately 22.94 ng/mL. Below this level, higher 25(OH)D was associated with lower odds of peripheral nerve damage, whereas above this level no statistically significant association was observed. However, this threshold was derived from the same dataset without internal validation, such as bootstrapping, and therefore should not be interpreted as a fixed cutoff or definitive threshold. In this cross-sectional population, patients with lower vitamin D status had a higher prevalence of peripheral nerve damage, but this finding should be considered exploratory and requires confirmation in prospective studies.

For carotid plaque, trend analyses indicated that decreasing 25(OH)D concentrations were generally related to greater odds of carotid plaque. Carotid plaque represents an important imaging indicator of subclinical atherosclerosis. Prior studies have reported that reduced vitamin D status is related to adverse carotid atherosclerosis phenotypes, including greater carotid intima-media thickness and increased likelihood of carotid plaque (26, 27). Our results are broadly aligned with these observations and suggest that reduced 25(OH)D concentrations may be associated with carotid atherosclerotic burden in patients with diabetes.

The relationship between 25(OH)D and carotid plaque may largely involve pathways related to atherosclerosis. Prior evidence suggests that vitamin D may enhance endothelial function, suppress monocyte and macrophage recruitment, limit cholesterol uptake and foam-cell formation, facilitate cholesterol efflux, and regulate macrophage polarization (28–30). Moreover, vitamin D/VDR signaling may influence RAAS activation, phenotypic transition of vascular smooth muscle cells, and vascular calcification (31, 32). These mechanisms provide pathophysiological support for the observed relationship between reduced 25(OH)D and carotid plaque.

For other outcomes, our findings suggest that reduced 25(OH)D concentrations may be related to greater urinary albumin excretion. Further restricted cubic spline and threshold effect analyses revealed a nonlinear association between 25(OH)D and log-transformed mALB, with a stronger association at lower 25(OH)D levels. This finding indicates that the relationship between 25(OH)D and renal injury-related indicators may be more pronounced in the setting of marked vitamin D insufficiency or deficiency. However, mALB primarily reflects abnormal urinary albumin excretion and does not fully capture overall renal function in patients with diabetes. The exploratory threshold of 11.48 ng/mL for mALB may suggest that the inverse association between 25(OH)D and renal injury-related indicators was more apparent at very low 25(OH)D concentrations in this dataset. However, this threshold was derived from the same dataset without internal validation, such as bootstrapping, and therefore should not be interpreted as a fixed cutoff or definitive threshold. This finding should be regarded as exploratory and hypothesis-generating rather than as a definitive cutoff or target value.

These identified thresholds should be distinguished from standard clinical definitions of vitamin D deficiency and insufficiency. Conventional cutoffs, such as deficiency defined as < 20 ng/mL and insufficiency as 20–30 ng/mL, are primarily used to classify vitamin D status, whereas the thresholds identified in this study were data-driven and outcome-specific inflection points for diabetes-related target organ damage indicators. The threshold of 22.94 ng/mL for peripheral nerve damage falls within the conventional insufficiency range, suggesting that the association with neural injury may be most evident in the lower vitamin D range. In contrast, the mALB threshold of 11.48 ng/mL is below the conventional deficiency cutoff, which may indicate that renal-related risk signals become more pronounced only at very low 25(OH)D concentrations. These discrepancies may reflect the characteristics of the hospitalized diabetes population, nonlinear risk relationships, limited sample size in some vitamin D categories, and potential model instability or statistical overfitting. Therefore, these thresholds should be considered exploratory and hypothesis-generating rather than alternative diagnostic cutoffs for vitamin D deficiency or insufficiency.

In addition, significant relationships of 25(OH)D with cerebral infarction and CHD were observed in the continuous-variable and sensitivity analyses. However, these results require cautious interpretation because event numbers were relatively small, the sufficiency group was limited in size, and stable effect estimates were not obtained in the categorical analyses. Validation in larger studies is therefore needed.

Collectively, lower serum 25(OH)D concentrations were associated with a greater likelihood of peripheral nerve damage and carotid plaque in patients with diabetes, and the mALB results also suggest a possible link with early renal injury. These observations raise the possibility that 25(OH)D may serve as an associated marker of peripheral nerve damage and carotid plaque in patients with diabetes. However, owing to the cross-sectional design and limited stability of some outcome estimates, 25(OH)D is better regarded as part of an integrated assessment than as an independent diagnostic indicator. Among patients with diabetes and a greater burden of diabetes-related complications, serum 25(OH)D levels may be interpreted together with disease duration, glycemic control, blood pressure, lipid levels, and other routine clinical indicators to describe the overall clinical profile. Current diabetes management increasingly emphasizes comprehensive assessment, individualized care, and the integration of treatment and prevention (33–35). However, because of the cross-sectional design of this study and the limited stability of some outcome estimates, the present findings should be interpreted as associative evidence only. Whether serum 25(OH)D provides incremental information beyond established clinical and biochemical indicators requires confirmation in prospective studies.

From an interventional perspective, our findings suggest that greater attention should be given to the assessment of vitamin D status in patients with diabetes and low 25(OH)D levels, including sun exposure, dietary intake, and supplementation. Confirmed deficiency should be corrected and monitored according to current guidelines and clinical practice standards. However, this was an observational study, and the findings do not support the conclusion that increasing 25(OH)D alone can reduce the risk of target organ damage. Any potential benefit of intervention still requires confirmation in prospective studies and randomized controlled trials.

Although previous observational studies and meta-analyses have reported associations between low vitamin D status and diabetic complications, causal evidence remains inconsistent. Recent Mendelian randomization studies did not support a clear causal effect of vitamin D on diabetic neuropathy, diabetic kidney disease, or diabetic retinopathy. Therefore, our findings should be interpreted as adding to existing cross-sectional evidence rather than establishing causality. The novelty of the present study lies in the simultaneous multi-outcome assessment of diabetes-related target organ damage indicators and the use of nonlinear and threshold analyses in a hospitalized Chinese diabetes population.

This study has several limitations. First, as an observational cross-sectional study, causal relationships cannot be established, and the findings should be interpreted as associations only. Second, this was a single-center, hospital-based study of hospitalized endocrine inpatients with diabetes; therefore, selection bias may exist, and the generalizability of the findings to community-dwelling, primary care, outpatient, or broader diabetes populations may be limited. Third, the relatively small number of participants in the 25(OH)D sufficiency group may have reduced statistical power and contributed to instability in some categorical regression estimates; therefore, categorical comparisons based on vitamin D status should be interpreted with caution. Because this was a retrospective cross-sectional study, no *a priori* sample size calculation was performed, and the study was not powered *a priori* for subgroup or categorical analyses. Therefore, some non-significant findings may reflect insufficient statistical power rather than a true absence of association. Fourth, although multiple potential confounders were adjusted for, unmeasured or residual confounding may remain (e.g., eGFR, statin use). Because patients receiving ACEIs or ARBs were excluded, the findings apply only to diabetic patients not treated with RAAS blockers. Generalizability to the broader diabetic population is limited. Seasonal variation in serum 25(OH)D concentrations was not fully evaluated, and detailed information on the dose, duration, and adherence to vitamin D supplementation was not consistently available; therefore, residual confounding related to seasonality and supplementation cannot be excluded. Vitamin D supplementation data were limited to in-hospital records; patients may have achieved sufficient levels through prior supplementation, diet, or sun exposure not captured in the medical record. Moreover, urinary albumin-to-creatinine ratio was not consistently available; therefore, mALB was used as a renal-related indicator, which may be affected by urine concentration.

## 5 Conclusion

Among patients with diabetes, lower serum 25(OH)D concentrations were associated with peripheral nerve damage, carotid plaque, and higher mALB levels. These findings support an association between serum 25(OH)D and indicators of diabetes-related target organ damage, but they do not establish causality or temporality. The reproducibility and longitudinal relevance of these associations require confirmation in larger prospective studies.

## Data Availability

The raw data supporting the conclusions of this article will be made available by the authors, without undue reservation.
