# Supplementary material for: Association of serum 25-hydroxyvitamin D levels with indicators of target organ damage in patients with diabetes: a cross-sectional study
Source: Front Endocrinol (Lausanne). 2026 Jun 29;17:1876261. doi: 10.3389/fendo.2026.1876261 (PMC13357216; doi:10.3389/fendo.2026.1876261)
Supplement: Supplementary file 1 [file DataSheet1.docx]

**Supplementary Materials**

**Supplementary Methods S1**. **Diagnostic criteria, outcome definitions, laboratory measurement methods, and handling of missing data**

**S1.1 Diagnostic criteria for diabetes**

For patients with a previous diagnosis of diabetes, the diagnosis was confirmed by reviewing medical history, antidiabetic medication records, and discharge diagnoses. For patients newly diagnosed with diabetes during the current hospitalization, diabetes was diagnosed according to the Guideline for the prevention and treatment of diabetes mellitus in China (2024 edition) issued by the Chinese Diabetes Society. In patients with typical symptoms of diabetes, diabetes was diagnosed if any of the following criteria were met: fasting venous plasma glucose ≥ 7.0 mmol/L, 2-hour venous plasma glucose ≥ 11.1 mmol/L during a 75-g oral glucose tolerance test, glycated hemoglobin A1c (HbA1c) ≥ 6.5%, or random venous plasma glucose ≥ 11.1 mmol/L. In patients without typical symptoms, the diagnosis required confirmation by two glycemic indices measured at the same time point or by repeated testing at different time points, excluding random plasma glucose. For patients with acute infection, trauma, or other stress conditions, diabetes was not diagnosed solely based on transient hyperglycemia during stress; glycemic status was reassessed after resolution of the stress condition, with concurrent HbA1c and glucose testing used to help distinguish stress hyperglycemia from diabetes.

**S1.2 Definitions of study outcomes**

(1) Peripheral neuropathy was assessed using nerve electrophysiological examination performed during hospitalization with an electromyography system manufactured by Shanghai Haishen Medical Instrument Co., Ltd. (NDI-094 model). The diagnosis of peripheral neuropathy was based on the diagnostic conclusion in the formal electrophysiological examination report issued by qualified clinicians or neurophysiology specialists. The electrophysiological diagnosis was generally based on abnormalities in nerve conduction parameters, including motor and/or sensory nerve conduction velocity, distal latency, compound muscle action potential amplitude, sensory nerve action potential amplitude, and F-wave responses, as interpreted according to the routine diagnostic standards of the clinical electrophysiology laboratory. In this retrospective study, peripheral neuropathy was defined as the presence of electrophysiological evidence of peripheral nerve damage in the formal report. The raw electrophysiological waveforms and parameters were not independently reinterpreted by the investigators.

(2) Carotid plaque was assessed by carotid ultrasonography performed during hospitalization using a GE LOGIQ E10s ultrasound system. The presence or absence of carotid plaque was determined according to the diagnostic conclusion in the formal carotid ultrasound report issued by experienced sonographers or ultrasound physicians. In routine institutional practice, carotid plaque was generally diagnosed as focal wall thickening protruding into the arterial lumen, usually with an intima-media thickness ≥1.5 mm or focal thickening at least 50% greater than the adjacent arterial wall. In this retrospective study, carotid plaque status was extracted from the formal ultrasound report rather than determined by independent remeasurement of carotid intima-media thickness, plaque thickness, or plaque morphology.

(3) Cerebral infarction was defined as a documented history or discharge diagnosis of cerebral infarction supported by cranial CT or MRI findings in the electronic medical record system. Both previous and current cerebral infarction documented in the medical record were considered.

(4) CHD was defined as documented coronary heart disease in the electronic medical record system, including previous myocardial infarction, angiographically confirmed coronary artery stenosis, prior percutaneous coronary intervention, prior coronary artery bypass grafting, or physician-diagnosed CHD recorded in the discharge diagnosis or medical history;

(5) mALB was measured using urine samples collected after admission and tested in the hospital clinical laboratory using routine standardized methods. In this study, mALB was used as an indicator of renal involvement. Because the urinary albumin-to-creatinine ratio (UACR) was not available for all participants in the current dataset, UACR was not used as the primary renal-related indicator. Given its skewed distribution, mALB was natural log-transformed before inclusion in the linear regression models. Before log transformation, mALB showed substantial skewness and kurtosis (skewness = 12.623; kurtosis = 166.465). After natural log transformation, the distribution was improved (skewness = 0.764; kurtosis = 0.880). The interpretation of mALB-related findings should therefore be cautious, as urinary microalbumin concentration may be affected by urine concentration.

Electrophysiological examinations, carotid ultrasonography, neuroimaging evaluations, and clinical diagnoses were performed as part of routine clinical care according to standardized departmental protocols, and formal reports were issued by qualified clinicians or specialists.

Because this was a retrospective study using clinical reports, formal blinding of outcome assessors to serum 25(OH)D levels and interobserver reliability assessment were not performed.

**S1.3 Measurement of serum 25(OH)D**

25(OH)D concentrations were measured using venous blood samples collected after admission. Serum 25(OH)D was measured in the hospital clinical laboratory using a chemiluminescence immunoassay method on a Zhengzhou Autobio chemiluminescence analyzer, model A6200. The assay was performed according to the manufacturer’s instructions, including instrument calibration, reagent use, and internal quality-control procedures. Analytical performance verification for the quantitative assay was conducted according to the Guideline for the verification of analytical performance of quantitative examination procedures (WS/T 408—2024) issued by the National Health Commission of the People’s Republic of China.

**S1.4 Flow diagram of patient selection and handling of missing data**

The patient selection process and handling of missing data are presented in **Supplementary Figure 1**. Patients were sequentially screened according to serum 25(OH)D measurement, age, diabetes type, prespecified clinical exclusion criteria, and availability of key covariates and primary outcome data. After excluding patients with missing key covariates or primary outcome data, the final complete-case analytic sample included 372 patients with no missing data for the variables included in the primary analyses.

**
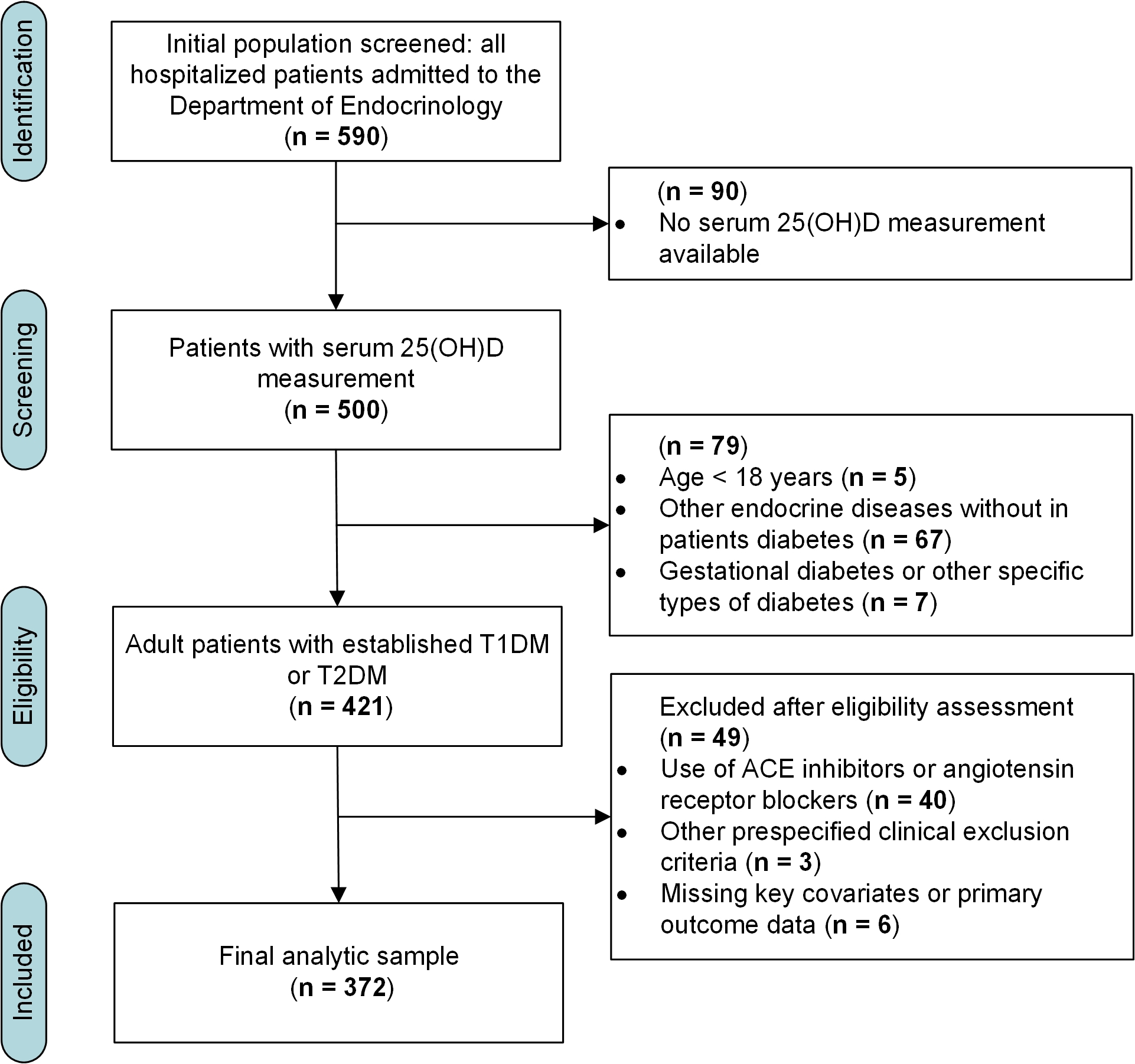
**

**Supplementary Figure 1** **Flow diagram of patient selection**

Note: Prespecified clinical exclusion criteria included severe hepatic or renal dysfunction, major active diseases, acute diabetic complications, and acute infection or stress conditions.

**S1.5 Model diagnostics**

Multicollinearity among serum 25(OH)D and covariates in the fully adjusted models (Model 3) was assessed using variance inflation factors (VIFs). For categorical variables with more than one degree of freedom, adjusted VIF values were calculated as GVIF^(1/(2×Df)). No substantial multicollinearity was detected, as all adjusted VIF values were below 5; therefore, all prespecified covariates were retained. (see **Supplementary File 2**)

For the linear regression model of ln(mALB), residual-versus-fitted and Q-Q plots were inspected to assess homoscedasticity and approximate normality of residuals. (see **Supplementary Figure 2**)


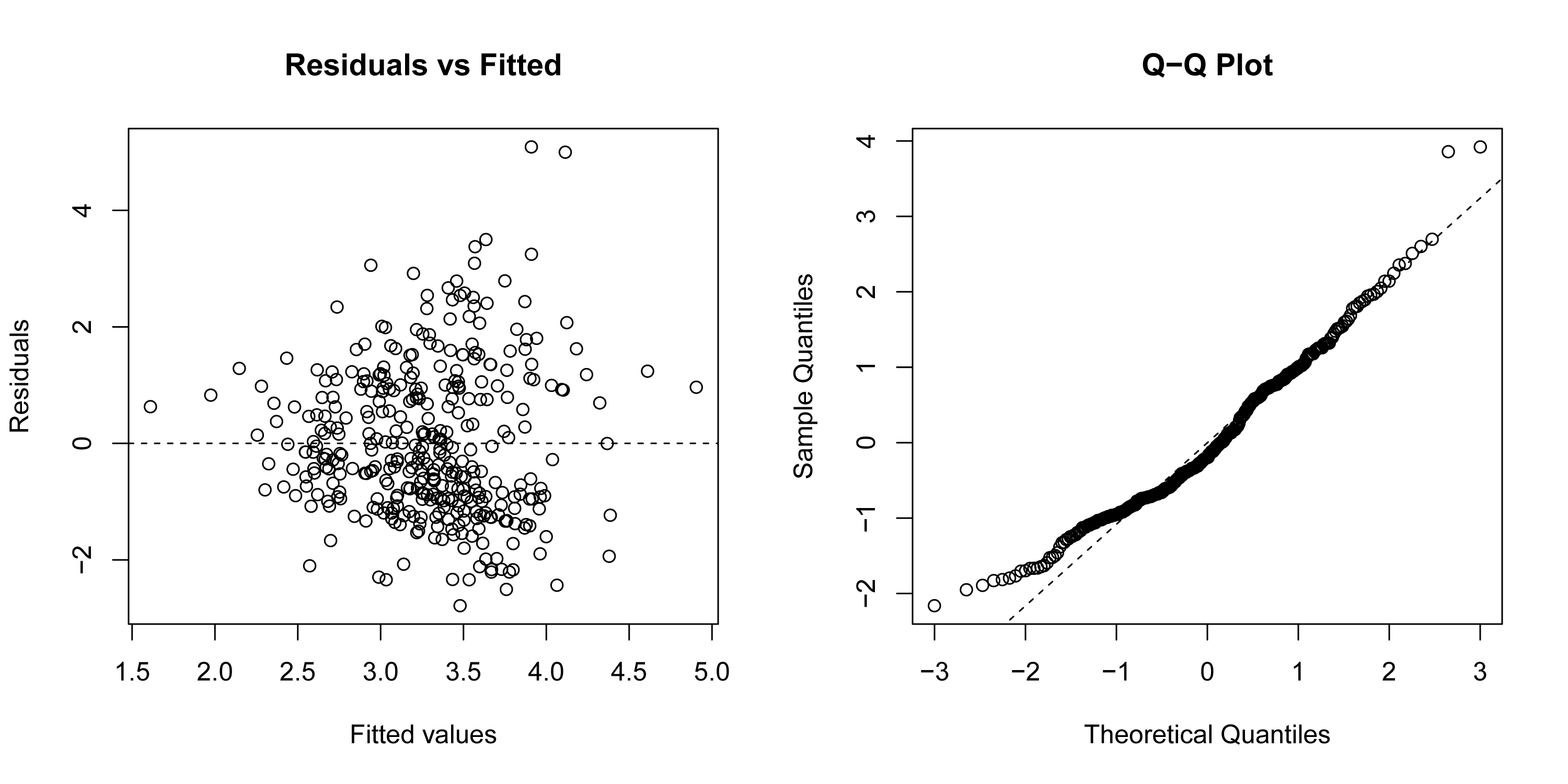


**Supplementary Figure 2** **Diagnostic plots for the fully adjusted linear regression model of ln(mALB)**

Note: Residual diagnostic plots for the fully adjusted linear regression model of ln(mALB). The residuals-versus-fitted plot was used to assess homoscedasticity and potential model misspecification, and the Q-Q plot was used to assess approximate normality of residuals. The residuals-versus-fitted plot showed no obvious nonlinear pattern, although mild heteroscedasticity and several positive residual outliers were observed. The Q-Q plot suggested approximate normality in the central distribution, with mild departures in the tails. mALB, urinary microalbumin.

For logistic regression models, apparent calibration was assessed using calibration plots, calibration intercepts, and calibration slopes. Calibration assessments were performed in the same dataset and were therefore interpreted as model diagnostics rather than external validation. (see **Supplementary Figure 3** )


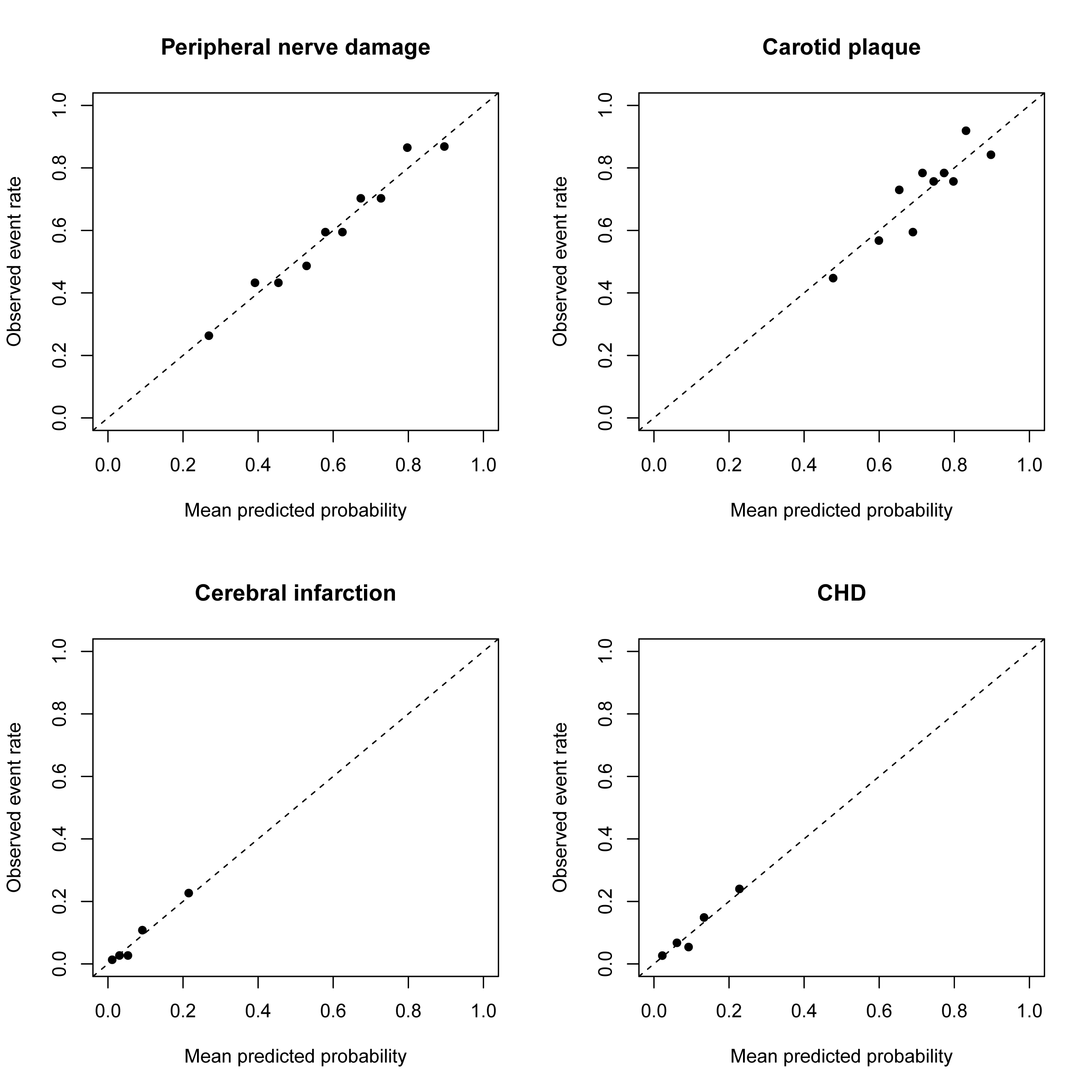


**Supplementary Figure 3**  **Calibration plots for the fully adjusted logistic regression models**

Note: Calibration plots for the fully adjusted logistic regression models of peripheral nerve damage, carotid plaque, cerebral infarction, and CHD. Participants were grouped according to predicted probabilities. Points represent the mean predicted probability and observed event rate within each group. The dashed line indicates perfect calibration. Apparent calibration plots showed generally acceptable agreement between predicted probabilities and observed event rates. For cerebral infarction and CHD, five groups were used because of the limited number of events. CHD, coronary heart disease.

For outcomes with limited event counts, including cerebral infarction and CHD, Firth penalized logistic regression was additionally performed as a sensitivity analysis.

**Supplementary Table Firth penalized logistic regression**

| Outcome | Method | *OR* | 95% *CI* | *P* value |
| --- | --- | --- | --- | --- |
| Cerebral infarction | Ordinary logistic regression | 0.896 | 0.816–0.971 | 0.013 |
| Cerebral infarction | Firth penalized logistic regression | 0.904 | 0.826–0.978 | 0.010 |
| CHD | Ordinary logistic regression | 0.902 | 0.835–0.966 | 0.006 |
| CHD | Firth penalized logistic regression | 0.909 | 0.843–0.972 | 0.004 |

Note: Firth penalized logistic regression was performed as a sensitivity analysis for outcomes with limited event counts. Models were adjusted for the same covariates as Model 3: age, sex, BMI, smoking, alcohol use, diabetes type, diabetes duration, hypertension, and NAFLD. *OR*s represent the change in odds per 1 ng/mL increase in serum 25(OH)D. CHD, coronary heart disease; *CI*, confidence interval; NAFLD, non-alcoholic fatty liver disease; *OR*, odds ratio.

**S1.6 Model formulas, simulation-based power analysis, and reproducibility**

For binary outcomes, including peripheral nerve damage, carotid plaque, cerebral infarction, and CHD, multivariable logistic regression models were used. The general model form was: logit[P(Y = 1)] = β0 + β1 × 25(OH)D + β2 × covariates

For mALB, linear regression models were fitted using natural log-transformed mALB as the dependent variable: ln(mALB) = β0 + β1 × 25(OH)D + β2 × covariates + ε

Serum 25(OH)D was analyzed both as a continuous variable, per 1 ng/mL increase, and as a categorical variable according to vitamin D status. In categorical analyses, the 25(OH)D sufficiency group was used as the reference category. No interaction terms were included in the main models.

Post hoc achieved power was estimated for the primary outcomes using simulation-based analysis with 1,000 repetitions. The simulations were based on the fully adjusted model (Model 3), observed effect estimates, current sample size, and observed covariate distribution. For binary outcomes, logistic regression models were used; for ln(mALB), linear regression was used. Achieved power was defined as the proportion of simulations in which the association between serum 25(OH)D and the outcome reached statistical significance at *α* = 0.05.

**Supplementary Table 1 Bonferroni sensitivity analysis for the five primary outcomes**

| Outcome | Original *P* value | Bonferroni-corrected threshold | Remained significant |
| --- | --- | --- | --- |
| Peripheral nerve damage | 0.001 | 0.01 | Yes |
| Carotid plaque | 0.002 | 0.01 | Yes |
| ln(mALB) | 0.002 | 0.01 | Yes |
| Cerebral infarction | 0.013 | 0.01 | No |
| CHD | 0.006 | 0.01 | Yes |

Note: Original *P* values were obtained from the fully adjusted Model 3 treating the exposure as a continuous variable for each of the five primary outcomes. The Bonferroni-corrected significance threshold was calculated as *α* = 0.05/5 = 0.01. Associations with original *P* values < 0.01 were considered statistically significant after Bonferroni correction. All *P* values were two-sided. ln(mALB), natural log-transformed microalbuminuria; CHD, coronary heart disease.

**Supplementary Table 2 Additional baseline characteristics according to serum 25(OH)D status**

| **Characteristics** | **25(OH)D levels** | | | | |
| --- | --- | --- | --- | --- | --- |
|  | **All (N = 372)** | **Sufficient (N = 25)** | **Insufficient (N = 133)** | **Deficient (N = 214)** | ***P* value** |
| **Cerebral infarction, n (%)** |  |  |  |  | 0.025 |
| No | 342 (91.94%) | 25 (100.00%) | 127 (95.49%) | 190 (88.79%) |  |
| Yes | 30 (8.06%) | 0 (0.00%) | 6 (4.51%) | 24 (11.21%) |  |
| **CHD, n (%)** |  |  |  |  | 0.087 |
| No | 332 (89.25%) | 25 (100.00%) | 121 (90.98%) | 186 (86.92%) |  |
| Yes | 40 (10.75%) | 0 (0.00%) | 12 (9.02%) | 28 (13.08%) |  |
| **Carotid plaque, n (%)** |  |  |  |  | 0.003 |
| No | 105 (28.23%) | 10 (40.00%) | 49 (36.84%) | 46 (21.50%) |  |
| Yes | 267 (71.77%) | 15 (60.00%) | 84 (63.16%) | 168 (78.50%) |  |
| **Peripheral nerve damage, n (%)** |  |  |  |  | < 0.001 |
| No | 151 (40.59%) | 10 (40.00%) | 73 (54.89%) | 68 (31.78%) |  |
| Yes | 221 (59.41%) | 15 (60.00%) | 60 (45.11%) | 146 (68.22%) |  |
| **TG, mmol/L** | 1.92 (1.18, 3.45) | 1.72 (1.16, 2.42) | 1.84 (1.05, 3.57) | 2.06 (1.29, 3.44) | 0.301 |
| **TC, mmol/L** | 5.04 ± 1.33 | 4.97 ± 1.19 | 4.86 ± 1.26 | 5.16 ± 1.38 | 0.114 |
| **LDL-C, mmol/L** | 3.33 ± 0.99 | 3.42 ± 0.98 | 3.25 ± 1.01 | 3.37 ± 0.98 | 0.473 |
| **Ca, mmol/L** | 2.34 (2.25, 2.41) | 2.33 (2.26, 2.40) | 2.34 (2.27, 2.40) | 2.34 (2.24, 2.42) | 0.898 |
| **UA, umol/L** | 313.86 (263.12, 386.10) | 297.70 (257.90, 345.20) | 305.20 (263.20, 372.70) | 324.30 (264.52, 395.18) | 0.390 |
| **HbA1c, %** | 9.90 (8.20, 11.60) | 10.50 (8.80, 12.20) | 9.60 (7.80, 11.90) | 9.95 (8.33, 11.40) | 0.450 |
| **C-peptide, ng/mL** | 1.92 (1.15, 2.86) | 1.94 (1.06, 2.98) | 1.75 (1.18, 2.70) | 2.12 (1.14, 2.98) | 0.524 |
| **Cr, umol/L** | 63.65 (52.20, 76.95) | 56.73 (31.80, 71.50) | 62.90 (52.90, 74.60) | 64.30 (52.82, 78.77) | 0.219 |
| **mALB, mg/L** | 18.00 (10.00, 57.77) | 25.10 (12.80, 64.82) | 15.30 (8.30, 50.90) | 18.55 (10.00, 58.52) | 0.236 |
| **Thyroid function, n (%)** |  |  |  |  | 0.330 |
| Abnormal | 41 (11.26%) | 5 (20.00%) | 14 (10.77%) | 22 (10.53%) |  |
| Normal | 323 (88.74%) | 20 (80.00%) | 116 (89.23%) | 187 (89.47%) |  |
| **VitD type, n (%)** |  |  |  |  | < 0.001 |
| No | 64 (17.20%) | 24 (96.00%) | 36 (27.07%) | 4 (1.87%) |  |
| VitD2 | 52 (13.98%) | 0 (0.00%) | 17 (12.78%) | 35 (16.36%) |  |
| VitD3 | 256 (68.82%) | 1 (4.00%) | 80 (60.15%) | 175 (81.78%) |  |
| **Treatment, n (%)** |  |  |  |  | 0.943 |
| OHA | 78 (20.97%) | 4 (16.00%) | 30 (22.56%) | 44 (20.56%) |  |
| Insulin | 72 (19.35%) | 4 (16.00%) | 26 (19.55%) | 42 (19.63%) |  |
| Both | 222 (59.68%) | 17 (68.00%) | 77 (57.89%) | 128 (59.81%) |  |

Note: Vitamin D supplementation refers to supplementation recorded in the electronic medical record during the current hospitalization. Patients categorized as having no vitamin D supplementation may still have achieved sufficient 25(OH)D levels through endogenous synthesis, dietary intake, prior unrecorded supplementation, or individual metabolic differences.

Values are presented as mean ± SD, median (Q1, Q3), or n (%). *P* values were derived from one-way ANOVA, Kruskal–Wallis test, or chi-square test, as appropriate;

CHD, coronary heart disease; TG, triglycerides; TC, total cholesterol; LDL-C, low-density lipoprotein cholesterol; UA, uric acid; HbA1c, hemoglobin A1c; Ca, serum calcium; mALB, microalbumin.

**Supplementary Table 3 Exploratory two-group sensitivity analysis for cerebral infarction and CHD according to vitamin D deficiency status**

| Outcome | Non-deficient group ≥ 20 ng/mL events/total (%) | Deficient group < 20 ng/mLevents/total (%) | *OR* (95% *CI*) | *P* value |
| --- | --- | --- | --- | --- |
| Cerebral infarction | 6/158 (3.80) | 24/214 (11.21) | 3.191 (1.230–9.789) | 0.011 |
| CHD | 12/158 (7.59) | 28/214 (13.08) | 1.829 (0.865–4.091) | 0.127 |

Note: The 25(OH)D sufficiency and insufficiency groups were combined into a non-deficient group (≥ 20 ng/mL) because no cerebral infarction or CHD events occurred in the 25(OH)D sufficiency group. The deficient group was defined as serum 25(OH)D < 20 ng/mL. Fisher’s exact test was used because of the limited number of events. *OR*s and 95% *CI*s were estimated from Fisher’s exact test and represent the deficient group compared with the non-deficient group. These exploratory results should be interpreted with caution because of the limited number of events and low statistical power. CHD, coronary heart disease; *OR*, odds ratio; *CI*, confidence interval.

In this exploratory two-group sensitivity analysis, cerebral infarction occurred in 6 of 158 participants (3.80%) in the non-deficient group and 24 of 214 participants (11.21%) in the deficient group *(OR* = 3.191, 95% *CI*, 1.230–9.789; Fisher’s exact *P* = 0.011). CHD occurred in 12 of 158 participants (7.59%) in the non-deficient group and 28 of 214 participants (13.08%) in the deficient group *(OR* = 1.829, 95% *CI*, 0.865–4.091; Fisher’s exact *P* = 0.127). We noted that these analyses were exploratory and limited by the small number of events.

**Supplementary Table 4 Distribution of ACEI/ARB use across vitamin D status groups in the expanded analysis set**

| **Variable** | **Sufficiency (n = 28)** | **Insufficiency (n = 148)** | **Deficiency (n = 236)** | ***P* value** |
| --- | --- | --- | --- | --- |
| ACEI/ARB use, n (%) | 3 (10.71%) | 15 (10.14%) | 22 (9.32%) | 0.895 |

Note: ACEI/ARB use was assessed after re-including the 40 patients who had previously been excluded because of ACEI or ARB use. The *P* value was calculated using Fisher’s exact test. ACEI, angiotensin-converting enzyme inhibitor; ARB, angiotensin receptor blocker.

**Supplementary Table 5 Sensitivity analysis for ln(mALB) after re-including ACEI/ARB users**

| **Analysis set** | **Model** | **N** | **Effect estimate for 25(OH)D** | ***P* value** |
| --- | --- | --- | --- | --- |
| Primary analysis set excluding  ACEI/ARB users | Model 3 | 372 | *β* = -0.035 (95% *CI*, -0.057 to -0.013) | 0.002 |
| Expanded analysis set re-including  ACEI/ARB users | Model 3 + ACEI/ARB use | 412 | *β* = -0.030 (95% *CI*, -0.053 to -0.007) | 0.010 |

Note: In the expanded analysis set, the 40 patients who had previously been excluded because of ACEI or ARB use were re-included, and ACEI/ARB use was additionally adjusted for as a covariate based on Model 3. Model 3 adjusted for age, sex, BMI, smoking, alcohol use, diabetes type, diabetes duration, hypertension, and NAFLD. *β* coefficients were estimated for ln(mALB) and represent the change in ln(mALB) per 1 ng/mL increase in serum 25(OH)D. ACEI, angiotensin-converting enzyme inhibitor; ARB, angiotensin receptor blocker; mALB, urinary microalbumin.

**Supplementary Table 6 Descriptive post hoc power estimates for the primary outcomes**

| Outcome | Model | Effect estimate in Model 3 | Sample size / events | Post hoc power estimate, % |
| --- | --- | --- | --- | --- |
| Peripheral nerve damage | Logistic regression | *OR* = 0.941 (95% *CI*, 0.905–0.976) | 221/372 events | 92.4 |
| Carotid plaque | Logistic regression | *OR* = 0.942 (95% *CI*, 0.906–0.977) | 267/372 events | 91.4 |
| ln(mALB) | Linear regression | *β* = -0.035 (95% *CI*, -0.057 to -0.013) | N = 372 | 87.9 |

Note: Post hoc power estimates were calculated using simulation-based analysis with 1,000 repetitions based on the fully adjusted model (Model 3), observed effect estimates, current sample size, and observed covariate distribution. For binary outcomes, logistic regression models were used; for ln(mALB), linear regression was used. These estimates are provided for descriptive purposes only and should not be interpreted as evidence that the study was adequately powered or that the sample size was justified. OR, odds ratio; CI, confidence interval; mALB, urinary microalbumin.

**Explanation of statistical methods for variables in Table 1**

(1) Shapiro–Wilk test results

| variables | *W* | *P* value |
| --- | --- | --- |
| Age | 0.988 | 0.003 |
| Diabetes duration | 0.849 | P < 0.001 |

(2) Histograms showing the distribution

| **A** | **B** |
| --- | --- |
| **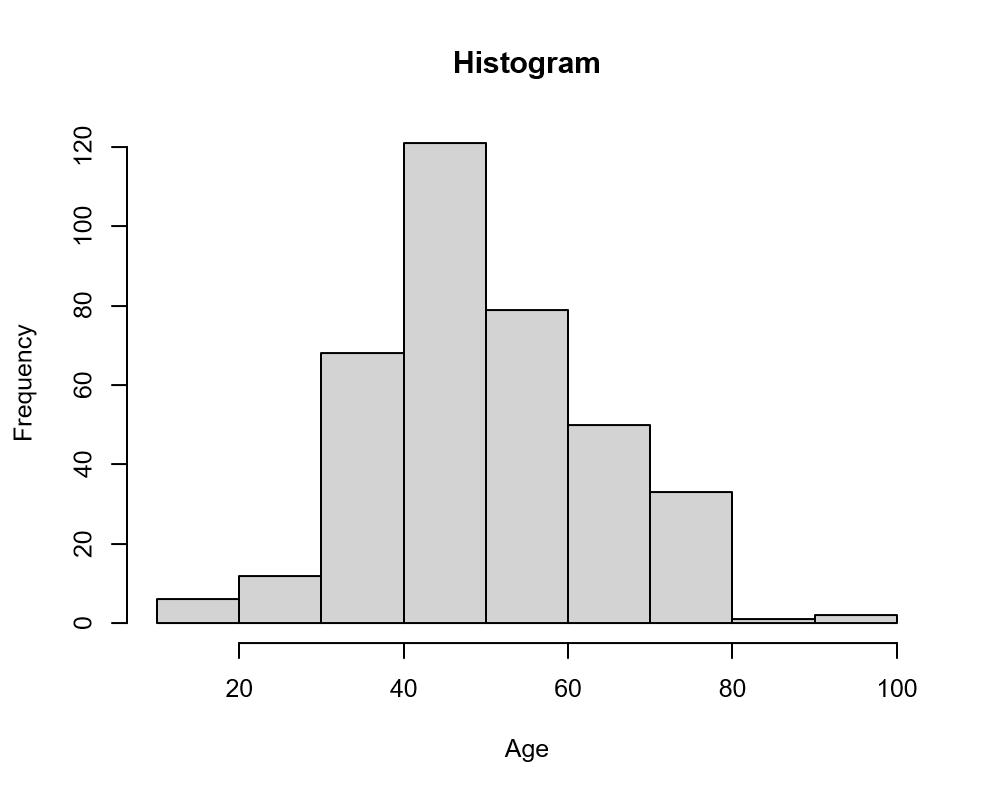** | **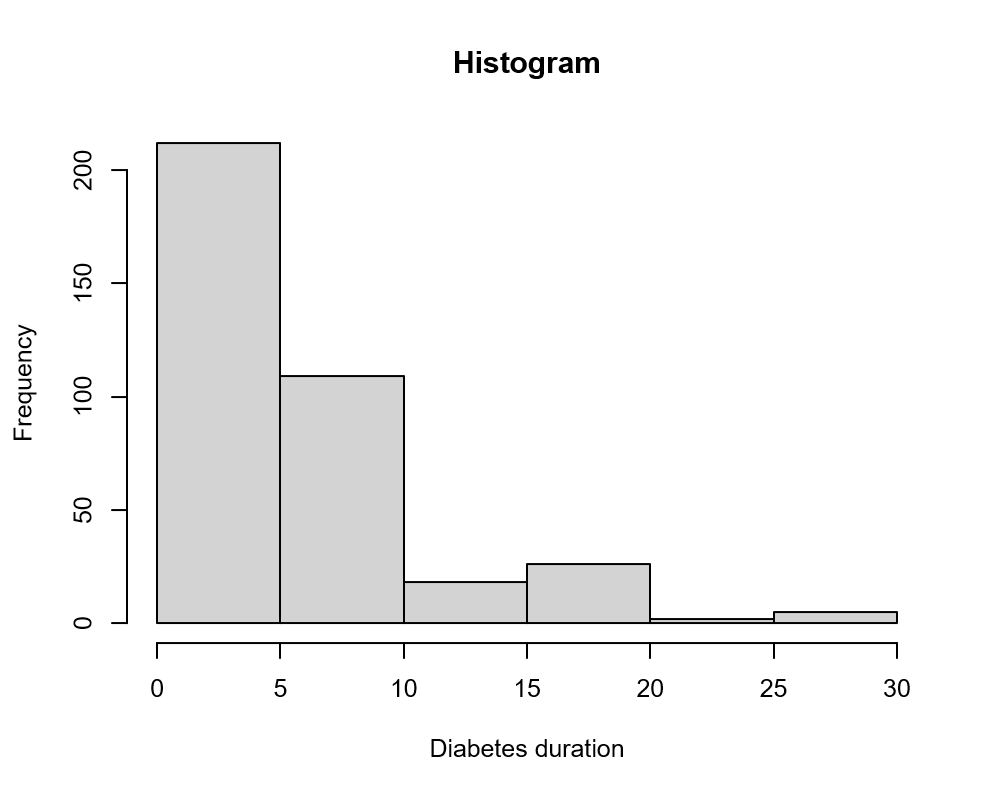** |

**Supplementary File 1 STROBE checklist for cross-sectional studies**

A completed STROBE checklist for cross-sectional studies is provided as a separate supplementary file. (see Supplementary File 1 uploaded separately)

**Supplementary File 2 Variance inflation factors for predictors included in Model 3**  (see Supplementary File 2 uploaded separately)

**Supplementary File 3 Full multivariable model estimates for all covariates** (see Supplementary File 3 uploaded separately)

Description: This supplementary file presents the complete regression estimates for all covariates included in the multivariable models evaluating the associations between serum 25(OH)D and diabetes-related target organ damage indicators. Logistic regression models were used for binary outcomes, including peripheral nerve damage, carotid plaque, coronary heart disease, and cerebral infarction. Linear regression models were used for log-transformed urinary microalbumin. For logistic regression models, results are presented as odds ratios (*OR*s) with 95% confidence intervals (*CI*s) and *P* values. For linear regression models, results are presented as regression coefficients (*β*) with 95% *CI*s and *P* values.

Model definitions: Crude model: unadjusted; Model 1: adjusted for age, gender, and BMI; Model 2: further adjusted for smoking history, alcohol consumption, diabetes type, and diabetes duration; Model 3: further adjusted for hypertension and NAFLD.

Notes: Serum 25(OH)D was analyzed as both a categorical variable and an ordinal trend variable. For categorical analyses, the vitamin D sufficient group was used as the reference category. The same covariate adjustment strategy was applied across outcomes. Age and gender were retained in all adjusted models.
