# Supplementary material for: Association of serum 25-hydroxyvitamin D levels with indicators of target organ damage in patients with diabetes: a cross-sectional study
Source: Front Endocrinol (Lausanne). 2026 Jun 29;17:1876261. doi: 10.3389/fendo.2026.1876261 (PMC13357216; doi:10.3389/fendo.2026.1876261)
Supplement: Supplementary file 2 [file SupplementaryFile1.pdf]

Supplementary File 1 STROBE checklist for cross-sectional studies

Manuscript: Association of serum 25-hydroxyvitamin D levels with indicators of target organ damage in patients with diabetes: A cross-sectional study

Section/Topic	Item No.	STROBE recommendation	Reported on page/line number(s)
Title and abstract	1a	Indicate the study's design with a commonly used term in the title or the abstract	Page 1, title; Page 1, Abstract-Methods.
Title and abstract	1b	Provide in the abstract an informative and balanced summary of what was done and what was found	Page 1-2, Abstract: Objective, Methods, Results, and Conclusions.
**Introduction**			
Background/rationale	2	Explain the scientific background and rationale for the investigation being reported	Page 2-3, Lines 56-85.
Objectives	3	State specific objectives, including any prespecified hypotheses	Page 3, Lines 86-88; Page 1, Abstract-Objective.
**Methods**			
Study design	4	Present key elements of study design early in the paper	Page 3, Lines 91-95; Page 4, Lines 98-100.
Setting	5	Describe the setting, locations, and relevant dates, including periods of recruitment, exposure, follow-up, and data collection	Page 3, Lines 91-95; Page 4-5, Lines 116-135.
Participants	6a	Give the eligibility criteria, and the sources and methods of selection of participants	Page 3, Lines 91-95; Page 4, Lines 101-115.
Variables	7	Clearly define all outcomes, exposures, predictors, potential confounders, and effect modifiers. Give diagnostic criteria, if applicable	Page 4-5, Lines 116-144; Page 5, Lines 145-155; Supplementary Methods S1.
Data sources/measurement	8*	For each variable of interest, give sources of data and details of methods of assessment (measurement). Describe comparability of assessment methods if there is more than one group	Page 4-5, Lines 116-144; Supplementary Methods S1.
Bias	9	Describe any efforts to address potential sources of bias	Page 3, Lines 91-95; Page 5, Lines 130-144; Page 13, Lines 376-395.
Study size	10	Explain how the study size was arrived at	Page 3, Lines 91-95; Page 4, Lines 101-115; Page 6, Lines 165-168; Page 13, Lines 383-386.
Quantitative variables	11	Explain how quantitative variables were handled in the analyses. If applicable, describe which groupings were chosen and why	Page 5, Lines 133-149; Page 5-6, Lines 145-155; Table 1; Table 2.
Statistical methods	12a	Describe all statistical methods, including those used to control for confounding	Page 5-6, Lines 145-164; Tables 2-5; Supplementary Methods S1.
Statistical methods	12b	Describe any methods used to examine subgroups and interactions	Not applicable: no prespecified subgroup or interaction analyses were conducted. Other exploratory analyses are described on Page 6, Lines 157-164, and Page 8, Lines 231-245.
Statistical methods	12c	Explain how missing data were addressed	Page 4, Lines 101-115; Page 5, Lines 141-144; Supplementary Methods S1.
Statistical methods	12d	If applicable, describe analytical methods taking account of sampling strategy	Not applicable: this was a hospital-based cross-sectional study without complex sampling.
Statistical methods	12e	Describe any sensitivity analyses	Page 6, Lines 157-164; Page 8-9, Lines 225-254; Table 5; Supplementary Tables 1, 3, and 4.
**Results**			
Participants	13a	Report numbers of individuals at each stage of study—eg numbers potentially eligible, examined for eligibility, confirmed eligible, included in the study, completing follow-up, and analysed	Page 4, Lines 101-115; Page 6, Lines 165-168. A total of 372 patients were included and analyzed.
Participants	13b	Give reasons for non-participation at each stage	Page 4, Lines 106-115. Exclusion criteria and reasons for exclusion are listed.
Participants	13c	Consider use of a flow diagram	Not used. Participant eligibility and exclusion criteria are described in the text.
Descriptive data	14a	Give characteristics of study participants (eg demographic, clinical, social) and information on exposures and potential confounders	Page 6, Lines 165-176; Table 1; Supplementary Table 2.
Descriptive data	14b	Indicate number of participants with missing data for each variable of interest	Page 4, Lines 101-115; Page 5, Lines 141-144; Supplementary Methods S1.
Outcome data	15*	Report numbers of outcome events or summary measures	Page 6-8, Lines 165-224; Tables 1-3; Supplementary Table 2.
Main results	16a	Give unadjusted estimates and, if applicable, confounder-adjusted estimates and their precision (eg, 95% confidence interval). Make clear which confounders were adjusted for and why they were included	Page 6-8, Lines 177-224; Tables 2 and 3. Model covariates are described on Page 5, Lines 150-155.

Section/Topic	Item No.	STROBE recommendation	Reported on page/line number(s)
Main results	16b	Report category boundaries when continuous variables were categorized	Page 5, Lines 133-135; Table 1; Table 2.
Main results	16c	If relevant, consider translating estimates of relative risk into absolute risk for a meaningful time period	Not applicable: this was a cross-sectional study; ORs and beta coefficients were reported without a follow-up time period.
Other analyses	17	Report other analyses done—eg analyses of subgroups and interactions, and sensitivity analyses	Page 8-9, Lines 225-254; Tables 4 and 5; Supplementary Tables 1, 3, and 4.
**Discussion**			
Key results	18	Summarise key results with reference to study objectives	Page 9, Lines 263-268; Page 14, Lines 396-401.
Limitations	19	Discuss limitations of the study, taking into account sources of potential bias or imprecision. Discuss both direction and magnitude of any potential bias	Page 13, Lines 376-395.
Interpretation	20	Give a cautious overall interpretation of results considering objectives, limitations, multiplicity of analyses, results from similar studies, and other relevant evidence	Page 9-13, Discussion; Page 11-12, Lines 326-341; Page 13, Lines 366-374; Page 14, Lines 396-401.
Generalisability	21	Discuss the generalisability (external validity) of the study results	Page 13, Lines 379-381.
**Other information**			
Funding	22	Give the source of funding and the role of the funders for the present study and, if applicable, for the original study on which the present article is based	Not applicable; no specific funding was received for this study, and there was no funder role.

Note: Page and line numbers refer to the revised manuscript provided for review. Items marked as not applicable are stated as such because the study was a hospital-based cross-sectional analysis without follow-up or complex sampling.
